# Remote and rural communities face inequalities in access to specialist palliative care, could telemedicine enhance care? A qualitative study of patient, carer and healthcare professionals’ experiences of video consultation

**DOI:** 10.1177/26323524251380632

**Published:** 2025-09-30

**Authors:** Lorelle Dismore, Katherine Frew, Donna Wakefield, Charlotte Bryan, Katherine Swainston

**Affiliations:** 1 Department of Innovation, Research and Development, Northumbria Healthcare NHS Foundation Trust, North Shields, UK; 2 Palliative Medicine Team, Northumbria Healthcare NHS Foundation Trust, North Shields, UK; 3 Specialist Palliative Care Team, North Tees and Hartlepool NHS Foundation Trust, Stockton-on-Tees, UK; 4 Population Health Sciences Institute, Faculty of Medical Sciences, Newcastle University, UK; 5 School of Psychology, Population Health Sciences Institute, Faculty of Medical Sciences, Newcastle University, UK; 6 Newcastle University Centre for Cancer, Newcastle University, UK

**Keywords:** palliative medicine, video consultations, patients, informal carers, qualitative method

## Abstract

**Background:**

Living in remote and rural areas is associated with worse health outcomes and poorer end-of-life care. Inequality in access to high-quality palliative care due to rurality is a worldwide problem. There is a need to evaluate potential ways, such as using telemedicine, to enhance palliative care and support patients to remain within their communities.

**Objective:**

To understand the experiences of patients, carers and specialist palliative care professionals to receive/deliver video consultations in a rural setting.

**Design:**

Qualitative semi-structured interviews with reflective thematic analysis.

**Methods:**

Patients, their informal carers (family/friend) and members of the community specialist palliative care team were invited to qualitative interview to explore their experience of using video consultations.

**Results:**

Four themes were generated including: (1) interpersonal communication, (2) enhanced provision with subthemes: physical distance and reducing travel and quick and convenient access to care, (3) flexible blended models of care and (4) organisational and practical barriers.

**Conclusion:**

In many situations, video consultations were felt to be beneficial to enable convenient and reliable access to specialist palliative care for patients living remotely. They are feasible, acceptable and practical for patients, their families and healthcare professionals. However, video consultations must be offered as an option to enhance care rather than replace in-person home visits, which are required in some situations. Further research is needed to explore how to ensure this increased accessibility is inclusive and supports disadvantaged older patients and those of lower socio-economic position.

## Background

Access to palliative care is a human right, with evidence that adequate access has multiple benefits including improving symptom control, quality of life and reducing admissions to hospital.^1–4^ An ageing population is associated with an increase in the prevalence of multiple long-term conditions and growing need for palliative care.^5^ People living in rural communities have poorer health outcomes than those living in urban areas, with a disproportionately older population contributing to a high need for palliative care in this setting.^6,7^ However, inequalities exist in accessing palliative care in rural areas, with rurality being a predicator of poorer end-of-life care.^8^

Nearly half (45%) of the world’s population live in geographically remote and/or rural areas.^9^ Living in remote areas with longer travel times is associated with increased Emergency Department visits and patients being less likely to receive specialist palliative care.^10^ Specialist palliative care can be delivered in the hospital, hospice or community. Most patients would prefer to receive care at home and avoid admissions to the hospital if possible. Delivery of specialist palliative care in the community setting is therefore vital; this usually includes outpatient clinic appointments, telephone appointments and visits to the patient’s home (Table 1 further describes specialist palliative care in the United Kingdom). Previous studies have identified that geographical distance can also be a barrier to accessing community palliative care, as it may be challenging for patients in poor health to travel to appointments and healthcare professionals may not be able to respond and offer home visits as quickly to remote areas due to long travel times, further complicated when adverse weather conditions occur.^11^

**Table 1.** Definition of specialist palliative care in the United Kingdom.^12^

Palliative care is an approach that aims to improve the well-being and quality of life of patients and their families facing life-limiting illness (across malignant and non-malignant disease). It involves holistic assessment and addressing physical, psychological, social and spiritual suffering. In the United Kingdom, all medical schools (46/46) and all nursing schools (90/90) have mandatory palliative care teaching, to equip all doctors and nurses to deliver generalist-level palliative care. Specialist palliative care services manage complex palliative care problems that cannot be managed by healthcare professionals who are not specialists in palliative care (such as primary care and other hospital-based specialists). Since 1987, Palliative Medicine has become recognised as its own medical specialty, for which physicians undertake a specific training programme. The specialist palliative care team also includes clinical nurse specialists who have undertaken additional training in palliative care. Many specialist palliative care teams also benefit from other members of the multidisciplinary team with additional training/experience in palliative care, such as specialist physios, occupational therapists, social worker, pharmacists. Specialist palliative care in the United Kingdom is free for patients to access and can be provided in hospital, at home or hospice. For this study, the specialist palliative care team refers to either a palliative medicine consultant, specialist palliative care nurse or specialist physiotherapist conducting a specialist assessment with a patient.

A recent systematic review of evidence-based models of rural palliative care identified a critical need to improve the provision of high-quality palliative care for people in rural areas, with cost-effective methods to sustain a connection between patients and specialist services needed.^13^ The use of digital technology, including telemedicine, is promising^14^ but requires further investigation if acceptable to an ageing population in rural communities.^15^

The COVID-19 pandemic led to accelerated adoption of technology and in many services’ rapid implementation of video consultations, to continue care whilst reducing transmission risk. However, this change has not always been sustained, with many returning to traditional face-to-face consultations. Studies have proposed that choice and flexibility are important and that a hybrid approach, such as giving telemedicine as an option would be beneficial to patients and healthcare professionals.^16^

People need equitable access to high-quality palliative care regardless of where they live. There is a need to evaluate ways, such as using telemedicine, to support patients to remain within their remote and rural communities.^17^ This study explored the experience of patients, carers and a specialist palliative care multidisciplinary team in the use of video consultation, as an option, with the aim of enhancing delivery of rural palliative care to reduce inequalities in access.

## Method

### Design

This study used a qualitative approach using semi-structured interviews to understand experiences of using video consultations for patients, informal carers (i.e. relative or friend providing unpaid care) and healthcare professionals (specialist palliative care doctors, nurses or physiotherapists).

### Authors’ philosophical positioning

An interpretivist paradigm with a subjective ontology was applied. The participants were viewed as people to make sense of their reality and reality being constructed in the context. An interpretivist epistemology enabled the use of qualitative methods to capture the participants’ experiences and opinions of the use of video consultations. Interviews facilitated exploration of the research questions to gain a deeper understanding of the phenomenon in the context of palliative care. The researcher (L.D.) was a female, health psychologist, experienced qualitative researcher and was independent from the palliative care clinical team.

### Setting

This study took place in Northumberland, where 46% of the population live in a rural environment, which contrasts with the United Kingdom average of only 18.5%. This is geographically one of the largest Trusts in England, providing services to more than 500,000 people, over more than 2500 mi^2^.

### Population

Participants included patients, informal carers and healthcare professionals. Patients had to be at least 18 years old, referred to the specialist palliative care service and able to provide consent. They were excluded if they were under 18 years old, lacked capacity to consent or if it was deemed inappropriate to use a video consultation (e.g. those who were imminently dying). The healthcare professionals were a member of the specialist palliative care team, who also consented to take part.

### Data collection

Members of the specialist palliative care team (doctor, nurse or physio) who would usually arrange either a home visit, telephone appointment or clinic appointment as part of their routine appointment, were (where appropriate) given the option to offer patients (and carers) an appointment via video consultation. The potential participants (patient and/or informal carer) were then invited to participate in the research by the clinical care team. If the patient/carer were interested in taking part in the study, then the clinical team (with permission) provided the contact information of the potential participants to the researcher. The researcher contacted the potential participant to provide further information and obtain informed witnessed consent. Sampling with patients and informal carers was convenience sampling over a 10-month recruitment and data collection period (June 2023–March 2024).

Following informed consent, a semi-structured interview regarding their experience of using the video consultation took place via telephone or video call, at a time convenient for the participant. Healthcare professionals were invited to participate in an interview up to a maximum of three times to allow for further reflections on their experience of the implementation of the video consultations. Interviews were audio-recorded and transcribed verbatim, and all the interviews lasted an average of 10 min. Interview schedules can be found in Appendix 1.

### Analysis

The semi-structured interviews were analysed using reflexive thematic analysis^18^ by one author (L.D.). Reflexive thematic analysis centralises on the researcher’s subjectivity, organic and recursive coding processes, emphasising the importance of deep reflection on, and engagement with, data as opposed to data saturation. The interviews conducted with patients and informal carers were analysed first, followed by the interviews with healthcare professionals. The initial steps involved familiarisation with the data by reading and re-reading each data transcript. Whilst reading over each data transcript, initial codes were used to highlight interesting aspects of the data. The codes were reviewed, refined and organised to look for patterns in the data. Once codes were established, they were arranged into potential themes. Themes were generated that represented patterns of shared meaning, underpinned by a central meaning-based concept. Reflections and themes were discussed and refined between two authors (L.D. and K.S.) throughout the write-up process, and consensus was reached in defining and naming the themes. Due to the commonality across the full data set, the themes represent the findings from the interviews conducted with all participants. Supporting quotations were selected that represent the themes.

### Ethical issues

This research received ethical approval from Wales Research Ethics Committee 4 (9 December 2022) and local Research and Development Approvals (16 June 2023). Informed witnessed consent was obtained from all the interviewees.

## Results

**Figure 1.**
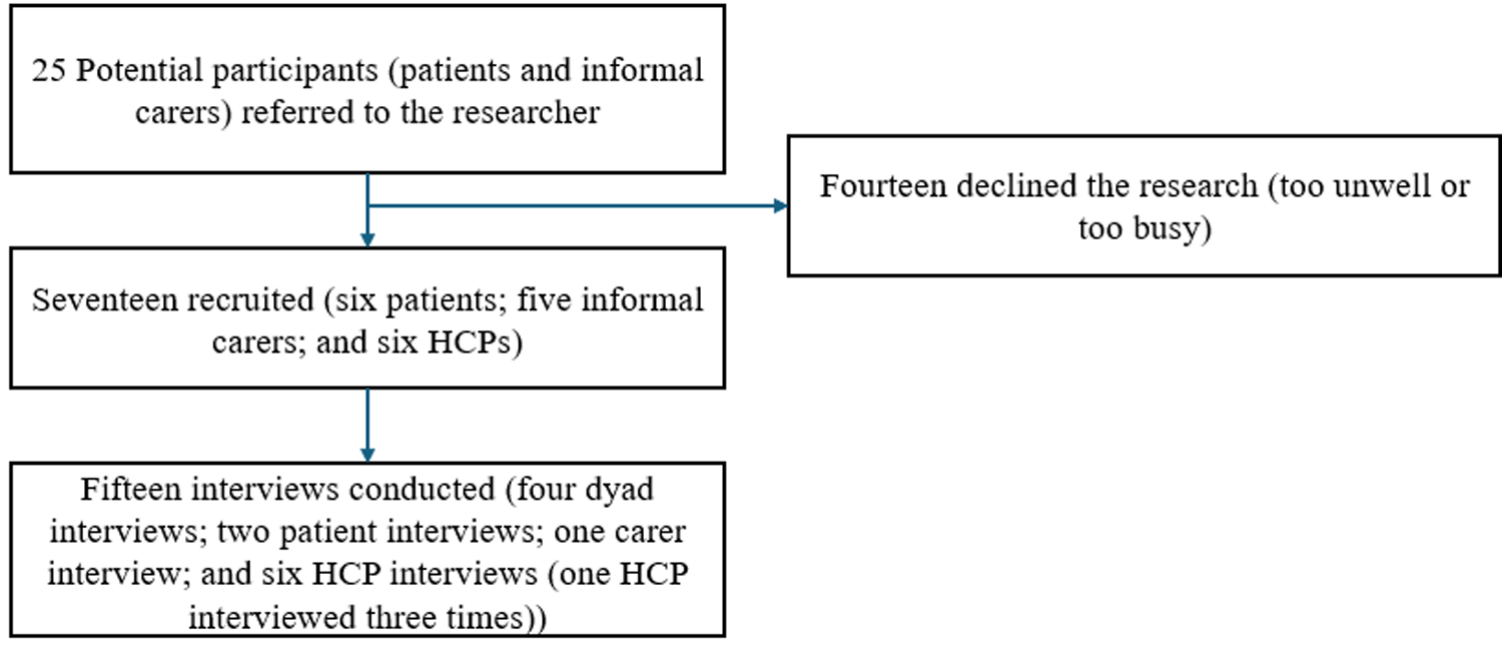
Participant recruitment flowchart.

A total of 17 participants were recruited, and 15 interviews were conducted (Figure 1). Of the 17 participants, 6 were patients (5 males and 1 female), 5 were informal carers (female spouses) and 6 were healthcare professionals (1 male and 5 females). Of the healthcare professionals, two were Community Nurse Specialist Macmillan Palliative Nurses, one Palliative Care Physiotherapist and three Palliative Care Consultants. Eleven interviews were conducted over a video call and four were conducted over a telephone call. Two patients and one carer were interviewed on their own, and four patients and their carer were interviewed together (dyad interview).

## Findings

Four themes were generated from the data: interpersonal communication, enhanced provision with subthemes: physical distance and reducing travel and quick and convenient access to care, flexible blended models of care and organisational and practical barriers.

### Theme 1: Interpersonal communication

A video consultation was advantageous compared to a telephone call, in providing clearer communication as the visual aspect of nonverbal communication would have otherwise been missed. The patients and informal carers reflected on how the video consultation facilitated meaningful discussions as they appreciated having the ability to ask questions with the healthcare professional via an easy communication method.

Better than a telephone conversation, it’s not quite as good as a person to person, it just seems a lot clearer and you can judge people’s, well doctors’ expressions I suppose. . .because you have a lot more meaningful discussion when you can actually see the person, talking to them rather than a voice somewhere in the ether. (Patient 06)

For the healthcare professional being able to see the patient during the video consultation meant that they were able to provide a detailed assessment of the patient compared to a telephone call.

When I’ve been up to the house you can sit there and you’re talking more generally about life and thing’s as well, whereas the video consultation seemed a little bit more practical. . . it’s just that getting used to it not feeling as personal. . . it just doesn’t seem as holistic as what it would do when you know you’re physically sitting with somebody. (HCP 02)

However, home visits were still valued for providing a holistic assessment of patients’ social circumstances that could be considered within management plans. Home visits were described as more personal.

I think with a face to face. . .I suppose you can pick up a little bit more of the body language and the sort of feel of the patient, whereas actually on a screen you still can’t quite feel that. . .but actually if someone in your house then you can definitely just pick up the vibes a bit more just a general feel of how things are. . .being able to see what’s going on in the house, and I think that’s a big part of our job is just being able to feel and understand what’s going on. (HCP 04)

Some of the participants (patients, informal carers and healthcare professionals) expressed a preference for an initial home visit prior to a video consultation to establish and build good rapport. Pre-existing relationships with patients and their families provided the healthcare professionals with the confidence to offer a video consultation.

I wouldn’t necessarily use it for an initial consultation, I think it’s important to have that sort of trust in getting to know people, unless there was a reason we couldn’t see them face to face, but I think it worked really well once you got to know the person and. . . I think it’s difficult to build the sort of rapport and the trust in those sorts of conversations when its online but as it was a follow up consultation, I’d already had quite a lot of conversations, got to know her, see what her sort of home situation was, I think that’s where it was really helpful for me. (HCP 05)

The interpersonal skills of the healthcare professional played a role in facilitating positive engagement and high satisfaction with the video consultation.

### Theme 2: Enhanced provision with subthemes: Physical distance and reducing travel and quick and convenient access to care

#### Time and travel saving

The video consultation was viewed by participants as simple to use and met patients and carers needs. It was convenient by not having to leave the home, commute from hospital or travel to and from rural areas.

I live very remotely. . .and I’m very conscious of the fact that people have to drive a really long way to come and see me and I know how pressed they are for time and how short staffed they are. . . I know they’ll be happier to just do more of us sitting from their desk where they’ve got all their own info, and I know that they’ll probably be doing their job with more confidence, more thoroughly because I know they don’t have to drive. . .fifty minutes to get to me. (Patient 05)

Informal carers also emphasised time saved travelling as a benefit of video consultations as opposed to travelling to a hospital facility for an appointment.

From where we live at times really it would save us a lot of travel time, and exhaustion than a face to face. (Wife of a patient)

The time saved travelling was beneficial for healthcare professionals in allowing for improved efficiency in workload and leaving work on time.

I see patients who are forty five minutes away, so that’s ninety minutes of time, of dead time. . .so you can do more work because you cut out a little bit of that time. . .and actually if you had an hour of your time back at the end of the day, does that mean you can do your documenting then and leave on time rather than leaving later. (HCP 01)

Support from informal carers during a video consultation was highly valued and important as they were active participants in the consultation. A healthcare professional emphasised how a video consultation would also allow to link up patients with their relatives at other physical locations in the future.

#### Quick and convenient access to care

Being able to have a video consultation was considered ideal for those who were shielding as it minimised face-to-face contact. It allowed for quick access to a healthcare professional when needing support. One carer emphasised how the video consultation reduced stress, as it allowed her to attend her relatives’ appointments without having to prepare medication around hospital visits.

He was quite stuck in the house. . .for him himself it was a lot easier. . . because. . .walking. . .caused a lot of pain. . .so much less stressful for him. . .I had to sort out medicines and pain relief. . .but like when it’s done from home it’s all at home, so there’s none, ‘oh you’ll need this, you’ll need that, you’ll need that’ because everything at home. . .he had his medicines at certain times, so like to take him out the house and that I would have to change them, which wasn’t beneficial for him or me, because then I’d have to think like how can we do this to make it all right for him. (Carer Wife of a patient)

A video consultation was practical. The healthcare professionals also emphasised the benefits of quicker accessibility of a professional while still being able to deliver effective clinical intervention. Video consultations avoided cancelling clinic and home visit appointments during to staff sickness. Video consultations allowed healthcare professionals to prepare in advance of in-person meetings.

You can get to speak to your patients and review them quicker, because ours live such rural areas so actually you spend quite a lot of time on the road but also like me today I’m full of cold, I can still do my job without going in and spreading my germs. . .and if you’re short staffed or you just quickly need to speak to someone but you want to do it over a video then actually it’s another options isn’t it? (HCP 04)

Not all patients and their informal carers preferred an in-person visit, and this was expressed in the context of quick and convenient access to care.

I wouldn’t have a preference really, would you? I don’t know, I could be quite happy with video, what you’re trying to do is have the best communication possible, I think over video you get that, and probably more quickly than if you were trying to do a face to face. (P02)

Being able to offer a video consultation as a method of communication was pragmatic as one example was provided whereby a patient’s appointment did not have to be cancelled and was replaced by a video consultation.

It was an unplanned discussion and doing it by video conference meant that I could fit it into my day whereas had I had to do a face to face that would have been challenging, had I done it as a phone call I wouldn’t have got the same information I got from the video call . . .there was very few staff around, but this woman needed to be seen. . .I could not have done a home visit. (HCP 06)

Video consultations offered enhanced care versus a telephone conversation. However, for some healthcare professionals, they preferred home visits due to the interpersonal aspect of consultations.

### Theme 3: Flexible blended models of care

Although video consultations were experienced successfully and preferred over a telephone consultation, participants reflected on how video consultations had to consider the context of the consultation.

Well I think it’s because the question I was asking didn’t need a face-to-face consultation, I think if it had been something a bit different it might have been that she would have had to come out to see me. . .if I was saying I’ve found a new lump or somethings changed it’s a bit difficult to say this is this, with me its more that they might want to come out and have a prod which you can’t do over a video consultation. (Patient 04)

The patient population was also emphasised as an important consideration for the appropriate method of communication. For example, a video consultation was described as being less appropriate for assessing patients with neurological problems and those with psychological distress.

It’s difficult because if I’ve been brought out for more of a psychological problem, and you do lose that little bit through the screen. (HCP 05)

Whereas a video consultation was viewed as being more helpful when patients only required advice versus physical care or examination. Concerns were addressed by patients and their carers understanding that they had the option of a home visit if needed due to the flexible approach of the healthcare professionals.

We weren’t only tied to a video; we have the confidence that if she saw that something was a matter or whatever she would make a visit. . .and that gives you confidence because I mean the care it’s been absolutely fantastic. (Wife of a patient)

The real reason why I’m most comfortable with it because I know were not doing this instead of something, it’s just to add another layer of what we can offer, you’re not going to take anything away. . . we have a choice as to whether we do this or we do a face to face. . .I think it’s going to be really useful for us as a rural team. (HCP 03)

A video consultation was considered beneficial as an extra optional method of communication, depending on the circumstances and participants would welcome them again in the future. They enabled for accessible and flexible models of care and the varied methods of communication meant that the models were responsive to individual’s needs.

### Theme 4: Organisational and practical barriers

The healthcare professionals suggested various practical considerations to consider for the future implementation of uptake of video consultations. The lack of rooms to hold the video consultation in privacy were of concern.

It will be looking at places where we can have something that’s a little bit more private. . .if we’re going to use this technology regularly. (HCP 03)

They didn’t anticipate other family members being present in the room during the video consultation and highlighted the need to establish relationships prior to the consultation. Yet, the inclusion of informal carers was also highly valued.

I think his wife was in the background. . .her sort of opinions across as well because he was doing it on his phone . . . I think if he’d been sitting with a laptop doing it and she could have participated, but then I didn’t ask her to sit where I could see her, I suppose that’s when you’re having a three-way conversation that could be a bit challenging. (HCP 02)

To overcome the challenges, training in how to deliver video consultations and written guidance with useful tips to provide to the healthcare professionals holding the video consultation and for the patients and informal carers would be beneficial, that is, instructions on positioning the phone. The healthcare professionals reflected on gaining confidence through continued use of video consultations.

Having a bit of a crib sheet for. . .these are the things you might want to think more about when you’re doing a video consultation, checking that your backgrounds okay, checking who’s in the room. . .an aide memoire of these are the things you might want to think about. . .I’m also gaining a little bit of confidence in it as well. . .how do I manage a consultation differently when it’s a different method. (HCP 06)

Two healthcare professionals suggested that a video consultation could be useful as a triage system to assess patient referrals. The ability to share scan results over a video consultation supported communication. A gradual culture change was required amongst the healthcare professionals to perform a consultation using a different method, and they would encourage their colleagues to use video consultations in the future.

## Discussion

For patients living in remote and rural communities, using video consultation enables quick and convenient access to specialist palliative care, which would usually be limited by the long travel time involved in a home visit or outpatient clinic. Patients living in rural communities also described situations, such as staff sickness, where they would usually struggle to access an alternative appointment. The advantage of video consultation was that they were able to have an appointment with an alternative member of staff with ease. This study builds on the evidence base that the use of video consultations is acceptable and valued by patients receiving palliative care, in many circumstances. As in previous studies, barriers of telehealth have included concerns regarding building relationships, and the quality of interpersonal communication. To potentially overcome this barrier, an initial in-person visit to build rapport prior to the option of video consultations for further appointments was preferable. Healthcare professionals emphasised a culture change towards accepting them as a method of communicating and gaining confidence through practice.

A recent systematic review reported that although many patients found tele-palliative care acceptable during the COVID-19 pandemic, they did not consider it a long-term solution when compared to in-person visits. However, some patients and carers felt video consultations may have a place beyond the pandemic, as it helped them cope better with their illness and would recommend it as a blended model in the future.^19^ Our study has been conducted in a post-pandemic context and has demonstrated acceptability of video consultations for direct patient care. Video consultations were appropriate for providing advice and guidance and patients and informal carers appreciated choice and flexibility in a hybrid approach.

This research has demonstrated a cost-effective method to sustain a connection between patients and specialist palliative care services using video consultations. The visual feature of video consultation was preferable to telephone as it enables patients to build interpersonal relationships with healthcare professionals over time^6^ and can enhance communication by expressing feelings, increased connectedness, caregiver support and improved advanced care planning.^15^ They have previously been used by clinicians, patients and caregivers to meet informational needs regarding pain and symptom management and medication use.^20^ The interviewees expressed concerns that video consultations may not be appropriate for individuals with psychological distress, however, they have previously been used to deliver cognitive interventions^21^ and are suitable for certain mental health conditions particular those with less severe forms.^22^ Adjustments to therapeutic practice can optimise video consultations and increase their effectiveness to address concerns.^23^ This multidisciplinary team support the use of video consultations for rapid access to patient care, but they also reflected on the added value of in-person home visits for the personal approach to care or when physical examination was necessary.

Digital health is growing in prominence in palliative care, and several technologies have been evaluated, reporting positive impacts on rural dwelling patient and caregiver well-being.^16^ Although video consultations are not suitable for all and/or for every scenario, they are valuable as an additional extra to increase flexibility and can enable those living in remote areas to receive rapid access to a review that they may not otherwise receive, while remaining in their rural and remote community.

There is a potential cost saving associated with video consultations in palliative care for clinicians, service providers, patients and caregivers, especially for those in rural settings.^24^ However, contextual factors such as geographical location and income have previously influenced perceptions of the added value of enhanced access^19^ and internet access remains an issue in rural and remote areas.^16^ There is therefore a risk of digital exclusion and the need for further research to explore if video consultations are accessible for patients of lower socio-economic status, which may in turn widen health inequalities. It is essential to invest in technology infrastructure, as well as training for healthcare providers, patients and carers to ensure equitable access in rural and remote areas.^16^

### Strengths and weaknesses

Recruitment to palliative care research can be challenging. This explorative study utilised convenience sampling to ensure successfully recruited across a large rural area and enable rapid feedback on the acceptability of video consultation as an option for delivery of community specialist palliative care. However, a larger study, with purposeful sampling of patient with a broad range of different diagnoses and demographics (ensuring inclusivity of older participants and those of lower socio-economic position) is warranted to explore not only general acceptability, but to whom and when. The transcripts were not returned to the patients and informal carers for member checking. Additionally, theoretical and conceptual frameworks could be applied, for example, technology and adoption theories aimed to understand and predict how individuals and organisations accept and integrate new technologies.

## Conclusion

Video consultations can be successfully implemented as an *option* for patients living in rural areas, potentially reducing inequalities in accessing timely and reliable specialist palliative care support within the community.

Remote video consultations can be acceptable and appropriate for patients receiving palliative care, but an initial in-person consultation to establish patient rapport prior to offering the option of video consultations for follow-up appointments was often preferred. Our findings suggest that video consultations are advantageous when compared to telephone appointments and can be effectively implemented in rural areas to enhance care. This must be considered only as an additional modality of care provision and not as a means of reducing or replacing home visits in community specialist palliative care services.

## Appendix

**Appendix 1:**

Topic Guide – Patient and/or relative 1. Can you tell me how easy or difficult you found setting up the video consultation? 2. Were there any technical issues? If so, how did you overcome them? Could you overcome them? 3. Did you know that video consultations were an option for you before now? 4. When (name of healthcare professional, or palcall) started the consultation did they introduce what they wanted to cover in the discussion? 5. Did you feel able to discuss what you wanted to? 6. Did you feel listened to? *NHCT only*: a If yes – was this in the usual way (name of healthcare professional) listens? b Was there anything different about the conversation or your ability to talk as you would want to? c If no – was this different to what you have experienced before from (name of healthcare professional) d Is there anything which would have made it easier? 7. Did you have any concerns about using video consultation? 8. If you were to be offered an option for a video consultation in the future, do you think you’d use it? If not, why? If yes – what do you find helpful about it? What do you like about the option? 9. If you/your loved one was to become more unwell, do you think that would alter your thinking about how useful the video consultation was? 10. Is there anything else you think we should know about using video consultation in community palliative care? Topic Guide – Healthcare professionals 1. Can you tell me how easy or difficult you found setting up the video consultation? 2. Did you have any concerns about using video consultation? 3. Were there any technical issues? If so how did you overcome them? 4. When you started the consultation did you introduce what you wanted to cover in the discussion? 5. Do you feel that the patient/carer understood? 6. What questions did the patient/carer ask you? 7. Did you feel able to discuss what you wanted to? 8. How would you describe the communication between yourself and the patient? *NHCT only*: *a Was this in the usual way as to as face-to-face consultation?* *b Was there anything different about the conversation or your ability to talk as you would want to?* *c Is there anything which would have made it easier?* 9. How do you feel about the use of video consultation in the future? 10. What did you find helpful about it? 11. What do you like about the option/what worked well? 12. What didn’t work so well and how could we improve video consultations? 13. What was missing from the video consultation? 14. Do you prefer video consultation or face-to-face visits? 15. Is there anything else you think we should know about using video consultation in community palliative care?
